# Matrix Gla Protein and Nitric Oxide Synthase-3 Genetic Variants in Chronic Kidney Disease and Their Relation with Cardiovascular Risk

**DOI:** 10.1155/2024/3850055

**Published:** 2024-08-30

**Authors:** G. Priyadarshini, Sreejith Parameswaran, Jayaprakash Sahoo, Sandhiya Selvarajan, Ananthakrishnan Ramesh, Medha Rajappa

**Affiliations:** ^1^ Department of Biochemistry Jawaharlal Institute of Postgraduate Medical Education and Research, Puducherry, India; ^2^ Department of Nephrology Jawaharlal Institute of Postgraduate Medical Education and Research, Puducherry, India; ^3^ Department of Endocrinology Jawaharlal Institute of Postgraduate Medical Education and Research, Puducherry, India; ^4^ Department of Clinical Pharmacology Jawaharlal Institute of Postgraduate Medical Education and Research, Puducherry, India; ^5^ Department of Radiodiagnosis Jawaharlal Institute of Postgraduate Medical Education and Research, Puducherry, India

## Abstract

**Background:**

Chronic kidney disease (CKD) is defined by gradual deterioration of renal parenchyma and decline of functioning nephrons. The risk of cardiovascular events is drastically increased in patients with CKD. This complicated link of CKD and cardiovascular disease (CVD) is not well understood till date.

**Objective:**

We aim to study the influence of genetic variants of matrix Gla protein (*MGP*) gene rs1800801, rs1800802, and rs4236 and nitric oxide synthase-3 (*NOS3*) gene rs1799983 and rs2070744 on the risk of CKD and its associated cardiovascular comorbidity in South Indian Tamils.

**Methods:**

One hundred and eighty-five CKD patients and 185 controls were recruited in this research. Flow-mediated dilatation (FMD) of brachial artery was measured ultrasonically. Circulating levels of MGP and nitric oxide (NO) were measured by ELISA. Genotyping was done by real-time PCR.

**Results:**

We observed a significant difference in the distribution of TT and CT genotypes of *NOS3* (rs2070744), indicating an increase in the risk of CKD. NO level was significantly decreased in CKD cases than controls. We also found a significant difference in the distribution of TTA and CCG haplotypes of *MGP* polymorphisms (1-rs4236; 2-rs1800801; 3-rs1800802) between the groups, indicating an increase in the risk of CKD. CT genotype of MGP (rs4236) and CT genotype of NOS3 (rs2070744) variants were found to be associated with decreased FMD, indicating endothelial dysfunction, the harbinger of CVD.

**Conclusion:**

We conclude that genetic variants of *MGP* and *NOS3* enhance the risk of CKD and its associated cardiovascular comorbidity in South Indian Tamils.

## 1. Introduction

Chronic kidney disease (CKD) is defined by the gradual deterioration of renal parenchyma and the reduction in the functioning nephrons. It has a prevalence of 17.2% in India [1]. Risk of cardiovascular disease (CVD) is drastically increased in CKD patients, particularly in those with end-stage renal disease (ESRD) [2]. In ESRD patients, vascular calcification (VC) advances quickly and hence is a reliable predictor of CVD [3]. A 60%–80% prevalence of mild to severe VC is seen in these patients [4]. CKD and CVD affect the occurrence and progression of each other in a complicated bidirectional manner [5].

Matrix Gla protein (MGP) is one of the body's most potent VC inhibitors [6]. It is a member of the GLA protein family that binds minerals and inhibits calcification [7]. Vascular smooth muscle cells in the artery wall are the primary producers of MGP, which has a molecular weight of 14 kD. In its carboxylated form, MGP acts as an inhibitor of calcification [8]. Many studies demonstrated the role of MGP on CVD risk in CKD patients, but there was minimal evidence in the literature that suggests a relationship between MGP and surrogate renal function indicators such as estimated glomerular filtration rate (eGFR) and proteinuria [9]. The *MGP* gene is located in the short arm of the 12th human chromosome. The human *MGP* gene has more than 120 single nucleotide polymorphisms (SNPs) identified. Due to their associations with CVD, rs1800801, rs1800802, and rs4236 are the most extensively studied polymorphisms. rs1800801 is a 5′ untranslated region variant, rs1800802 is a 2 kB upstream variant, and rs4236 is a missense variant that causes the substitution of threonine for alanine [10].

Endothelial dysfunction has been found to be much more common in CKD patients, and it positively correlates with CKD severity [11]. It has been linked to an elevated risk of the development and progression of CVD and is a crucial component of all phases of atherosclerosis [12]. The primary functional aspect of endothelial dysfunction is a reduction in endothelium-dependent vasodilatation. This process is primarily controlled by synthesizing and releasing nitric oxide (NO) in endothelial cells in response to shear stress. Endothelial dysfunction prevents the adhesion and migration of leukocytes to arteries, platelet aggregation, and the proliferation of smooth muscle cells, all of which are crucial factors in the development of atherosclerosis [13]. NO is synthesized from the metabolism of L-arginine by endothelial nitric oxide synthase (eNOS). Chronic inhibition of NOS has been found to be associated with CKD. Multiple studies on animals have shown that chronic NOS suppression under experimental conditions results in systemic and glomerular hypertension, glomerular ischemia, glomerulosclerosis, tubulointerstitial damage, and proteinuria [14]. eNOS gene is located in chromosome 7q35-36 [15]. Several genetic variants are identified in this gene, among which rs1799983 and rs2070744 have been given much attention due to their association with CVD. rs1799983 is a missense variant that leads to the substitution of aspartic acid for glutamic acid. rs2070744 is an intron variant [16].

Our research group has previously explored the interrelationship between endothelial dysfunction and CKD severity [11]. As an extension of this work, we undertook to explore the relationship between genetic variants of *MGP* (rs1800801, rs1800802, and rs4236) and *NOS3* (rs1799983 and rs2070744) genes on the risk of CKD and its associated cardiovascular comorbidity in South Indian Tamils. Patients with CKD comprise a heterogeneous population with various mechanisms of CVD progression. Since there are a number of interrelated factors, CKD patients both those with and without diabetes have a higher risk of developing CVD. The significance of personalized medical care and risk assessment for CKD patients is important because of this heterogeneity. Hence, we wanted to study a homogenous group of nondiabetic CKD patients in this research.

## 2. Methods

### 2.1. Study Participants

One hundred and eighty-five nondiabetic, pre-dialysis South Indian Tamil CKD patients and 185 age, gender, and ethnicity-matched healthy control volunteers were enrolled in the study at a tertiary care centre in South India. This study (Project number: JIP/IEC/2019/0546) was approved by the Ethics Committee and was in concordance with the Indian Council of Medical Research (ICMR) ethical guidelines for biomedical research involving human participants, 2017. After describing the study's procedure to all participants, written informed consent was obtained from them. South Indian nondiabetic pre-dialysis CKD patients with an age range of 18–70 years attending the nephrology clinic were included in the study. Pregnant or lactating women, patients with immobilization of 3 months, inherited diseases of bone, inherited causes of CKD, malignancy, neoplasia, infections, coronary artery disease, peripheral artery disease, and cerebrovascular disease were excluded from the research. Tamil families that had lived in Tamil Nadu and Pondicherry for at least three generations were included as study participants in this study to maintain homogeneity in the sample.

### 2.2. Sample Size Calculation

The sample size was calculated using the minor allele frequencies of the genetic variants under consideration. The sample size was calculated at a 5% significance level with 90% power for the estimated disease prevalence of 17.2% [1]. The minor allele frequency of 0.17 for *NOS3* (rs1799983) (https://www.ncbi.nlm.nih.gov/snp/rs1799983) yielded a higher sample size, and hence it was considered. The estimated sample size was 185 in each group (CKD patients and controls). The sample size for this genetic study was calculated using the CaTS Power Calculator Software (Power Calculator for Genetic Studies, compiled and published by the Centre for Statistical Genetics, Michigan University, USA).

### 2.3. Assessment of Endothelial Dysfunction by Flow-Mediated Dilatation

Flow-mediated dilatation (FMD) of the brachial artery was performed using a Prosound alpha six color Doppler ultrasound scanner (Aloka, Japan) and a 9 MHz linear array transducer. Before the procedure, the participant was told to fast for 6 to 8 hours. The brachial artery was scanned with the participant in supine posture. For a minimum of a day before the procedure, the participants were told not to use cigarettes, drink alcohol, or take any medicines that could impair endothelial function. The probe was positioned across the brachial artery of the left upper arm in a longitudinal portion just above the cubital fossa. The brachial artery's echogenic intimal layers were displayed, and a baseline measurement of the brachial artery's width between the near and distant intimal layers was taken. The average of three observations was used to calculate the baseline diameter. Around the upper forearm, a blood pressure cuff was tied, and it was inflated for 5 minutes to maintain a pressure 50 mmHg higher than the participant's systolic blood pressure. The cuff was removed after 5 minutes. Two minutes after the cuff was removed, the brachial artery's width was measured above the cubital fossa. The post-FMD diameter was calculated using the average of the three values.

The given formula was used to measure FMD, also known as proportional FMD:

(1) $$\begin{array}{c} \text{FMD}=\left(\frac{\left(\text{post}-\text{FMD}\;\text{diameter}-\text{baseline}\;\text{diameter}\right)}{\text{baseline}\;\text{diameter}}\right)*100. \end{array}$$

### 2.4. Evaluation of Metabolic Syndrome and Estimation of MGP and NO Levels

Metabolic syndrome was evaluated by the National Cholesterol Education Program-3 (NCEP-3) criteria [17]. Plasma from the blood samples were separated by centrifugation after being collected in the EDTA tube. The plasma samples were then kept at −80°C until they were evaluated. Using commercially available ELISA kits (Krishgen Biosystems, India), plasma levels of MGP and NO of all the research participants were measured in accordance with the manufacturer's instructions.

### 2.5. Genotyping

From the research participants, 5 mL of blood was collected. Using a commercially available DNA extraction kit (QIAmp DNA Blood mini kit, Qiagen, Germany), genomic DNA was isolated. Prior to use in genotyping, DNA concentration was quantified and diluted to 50 ng/*μ*l, and stored at −80°C. SNPs of *MGP* (rs1800801, rs1800802, and rs4236) and *NOS3* (rs1799983 and rs2070744) were selected using the candidate gene approach. Genotyping of *MGP* (rs1800801, rs1800802, and rs4236) and *NOS3* (rs1799983 and rs2070744) polymorphisms were done by a predesigned allelic discrimination assay using TaqMan probe‐based 5-nuclease chemistry using Quant Studio 5 Real-time PCR system (Thermofisher scientific, USA).

### 2.6. Statistical Analysis

The genotypes and allele frequencies of the variants were calculated using the direct gene counting method. Chi-square test was used to perform the Hardy–Weinberg equilibrium (HWE), which compares the observed and expected allele frequencies. For examining the genotype and allele frequency between the patients and controls and also to generate odds ratio and 95% confidence intervals, Chi-square test or Fischer's exact test was used. Chi-square test or Fischer's exact test was used to access the association between frequency of genotypes with phenotypes of CKD (gender, family history of kidney disease, stages of CKD, and metabolic syndrome) within the patient population. Results were considered significant when the two-tailed *P* value was <0.05. The effect of *MGP* and *NOS3* gene polymorphisms on their respective plasma concentrations and FMD were examined by the Kruskal–Wallis test. Haplotype frequencies and linkage disequilibrium (LD) between pairwise SNPs within the *MGP* and *NOS3* genes were measured using *D*′ and *r*^2^ statistics with Haploview software version 2.4 (Cambridge, Massachusetts). SNP-SNP interaction between the different genes was assessed using R package SNP Interaction Pattern Identifier (SIPI). SIPI tests 45 biologically meaningful interaction patterns for SNP-SNP interactions by considering three SNP inheritance modes (additive, dominant, and recessive). IBM SPSS Statistics (IBM Corp. Released 2011. IBM SPSS Statistics for Windows, Version 20.0), GraphPad prism version 8 (San Diego, California, USA), and R programming software version 4.3.3 for Windows was used for the statistical analysis.

## 3. Results

### 3.1. Demographic and Clinical Characteristics of the Study Participants

One hundred and eighty-five patients with CKD and 185 age and gender-matched healthy controls were recruited in the study. The demographic characteristics of the CKD patients and controls are given in Table 1. Out of the 185 cases, 26 CKD patients had metabolic syndrome. The median age of patients during enrolment was 53 years, and the median duration of the disease at the time of recruitment was 43 months. Among the 185 CKD cases, 100 were in stage 4, 79 were in stage 3, and 6 were in stage 2 of the disease. While comparing baseline biochemical parameters, serum creatinine, urea, total cholesterol, very low-density lipoprotein, low-density lipoprotein, triglycerides, high-density lipoprotein, and phosphorous levels were observed to be significantly higher in the cases compared to controls. Serum calcium, total protein, albumin levels, and flow-mediated dilatation of brachial artery levels were found to be significantly reduced in the CKD cases compared to controls (Supplementary Table 1).

### 3.2. Association of *MGP* Polymorphisms with CKD Susceptibility

When we compared the genotype and allele frequencies of *MGP* SNPs (rs1800801, rs1800802, and rs4236) between patients with CKD and controls, we found no significant difference. The case and control genotypes were concordant with the HWE. The observed frequency of an ancestral genotype (CC) of *MGP* (rs1800801) polymorphism was 92 (49.7%) and 83 (44.9%) in cases and controls, respectively. The mutant genotype (TT) frequency in the cases and controls were 19 (10.3%) and 17 (9.2%), respectively (Table 2). These results were also confirmed with genetic model analysis, which showed that in the South Indian Tamil population, rs1800801 is not a genetic risk factor for developing CKD (Supplementary Table 2). In addition, we divided the patients into groups according to their clinical characteristics such as gender, family history of kidney disease, CKD stages, and presence of metabolic syndrome. However, we found no association between the phenotypes and genotypes of rs1800801 (Supplementary Table 3).

In cases and controls, the observed frequency of the ancestral genotype (AA) of *MGP* (rs1800802) polymorphism was 93 (50.3%) and 83 (44.9%), respectively. The frequency of the mutant genotype (GG) was 17 (9.2%) in the patients and 12 (6.5%) in the controls (Table 2). These findings were further confirmed by genetic model analysis, which revealed that rs1800802 was not a risk factor for developing CKD in South Indian Tamils (Supplementary Table 2). We found no correlation between clinical phenotypes and the *MGP* SNP rs1800802 after dividing the cases based on clinical phenotypes (Supplementary Table 4).

The observed frequency of the ancestral genotype (TT) of *MGP* (rs4236) polymorphism was 50 (27.0%) and 46 (24.9%) in cases and controls, respectively. The frequency of the mutant genotype (CC) was 42 (22.7%) in the patients and 42 (22.7%) in the controls (Table 2). These results were also confirmed using the genetic model analysis, which showed that in the South Indian Tamil population, rs4236 was not a genetic risk factor for developing CKD (Supplementary Table 2). In addition, we divided the patients into groups according to their clinical characteristics, and we did not find any association between the phenotypes and genotypes of rs4236 (Supplementary Table 5). Among the polymorphisms of *MGP*, FMD was significantly reduced in the CT genotype of rs4236 compared to CC and TT genotypes, indicating an endothelial dysfunction and hence risk of CVD (*P* value = 0.041) (Table 3).

**Figure 1:**
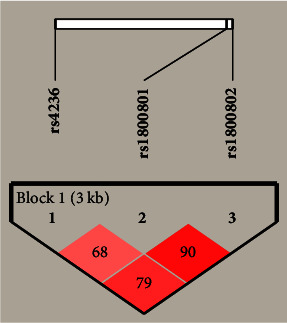
The LD plot of the *MGP* gene polymorphisms (rs4236, rs1800801, and rs1800802). Each square shows the pairwise LD relationship between two SNPs, and the values inside the square denote *D*′ value. The color gradient from red to white reveals higher to lower LD, and the color scheme is based on the standard *D*′/LOD (logarithm of odds).

Linkage disequilibrium analysis revealed a strong linkage disequilibrium between rs1800801 and rs1800802 (*D*′ = 0.9, LOD = 15.84, *r*-squared = 0.159). We also found a strong linkage disequilibrium between rs4236 and rs1800802 (*D*′ = 0.79, LOD = 23.01, *r*-squared = 0.25) and moderate linkage disequilibrium between rs4236 and rs1800801 (*D*′ = 0.68, LOD = 20.48, *r*-squared = 0.22) (Figure 1). Eight different haplotypes were derived from these three SNPs. However, only six haplotypes with frequencies greater than 1% were present. We found a statistically significant difference in the distribution of the TTA and CCG haplotypes (1-rs4236; 2-rs1800801; 3-rs1800802) in the two groups, indicating an increase in the risk of CKD (Table 4).

**Figure 2:**
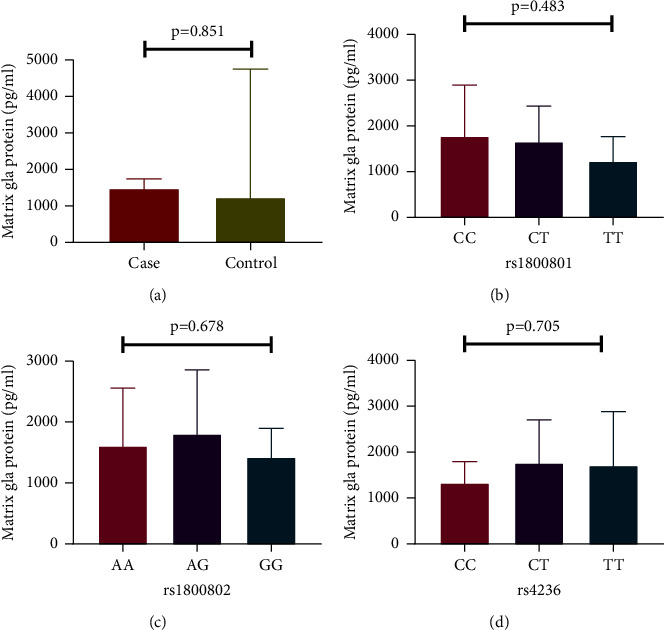
Circulating plasma MGP levels between CKD cases and controls and the influence of *MGP* polymorphisms (rs1800801, rs1800802, and rs4236) on MGP levels.

When we compared the plasma MGP levels between CKD patients and controls, we did not find any significant difference (1476.56 pg/mL (1196.63–1737.63) vs. 1229.99 pg/mL (991.04–4746.38), *P*=0.851) (Figure 2). The genotypes of *MGP* polymorphisms (rs1800801, rs1800802, and rs4236) did not significantly affect stage of CKD, metabolic syndrome (Supplementary Tables 3, 4, and 5), and plasma MGP levels (Figure 2).

### 3.3. Association of *NOS3* Polymorphisms with CKD Susceptibility

When we compared the genotype and allele frequencies of *NOS3* (rs1799983) between patients with CKD and controls, it yielded no significant difference. The case and control genotypes were concordant with the HWE. Observed frequencies of the ancestral genotype (TT) of *NOS3* (rs1799983) in patients and controls were 6 (3.2%) and 7 (3.8%), respectively. A total of 130 patients (70.3%) and 139 controls (75.1%) carried the mutant genotype (GG) (Table 5). This result was further verified by genetic model analysis, which indicated that rs1799983 was not a genetic risk factor for developing CKD in the South Indian Tamil population (Supplementary Table 6). After partitioning the cases based on clinical phenotypes (Supplementary Table 7), we observed no significant differences between the groups for this genetic variant.

We found a significant difference in the genotype frequency of ancestral genotype CC of *NOS3* (rs2070744), which was 5 (2.7%) and 16 (8.7%) in cases and controls, respectively, indicating its protective effect against CKD development. Mutant genotype (TT) frequency in the cases and controls were 115 (62.2%) and 109 (58.9%), respectively (Table 5). In recessive model analysis, TT and CT genotypes were found to be risk genotypes for CKD (Supplementary Table 6). We find no correlation between clinical phenotypes and the *NOS3* SNP rs2070744 after dividing the cases based on clinical phenotypes (Supplementary Table 8). Among the polymorphisms of *NOS3,* FMD was significantly reduced in the CT genotype of rs2070744 compared to CC and TT genotypes, indicating an endothelial dysfunction and hence risk of CVD (*P* value = 0.01) (Table 3).

**Figure 3:**
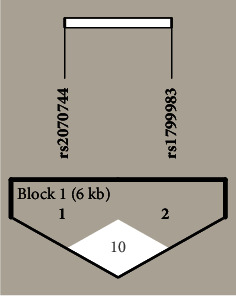
The LD plot of the *NOS3* gene polymorphisms (rs2070744 and rs1799983). Each square shows the pairwise LD relationship between two SNPs, and the values inside the square denote *D*′ value. The color gradient from red to white reveals higher to lower LD, and the color scheme is based on the standard *D*′/LOD (logarithm of odds).

When we studied the linkage disequilibrium between rs2070744 and rs1799989 polymorphisms of the *NOS3* gene, we found a weak linkage disequilibrium (*D*′ = 0.1, LOD = 0.58, *r*-squared = 0.006) (Figure 3). We did not find any significant difference in the distribution of the haplotypes of *NOS3* genetic variants between CKD cases and controls (Table 6).

**Figure 4:**
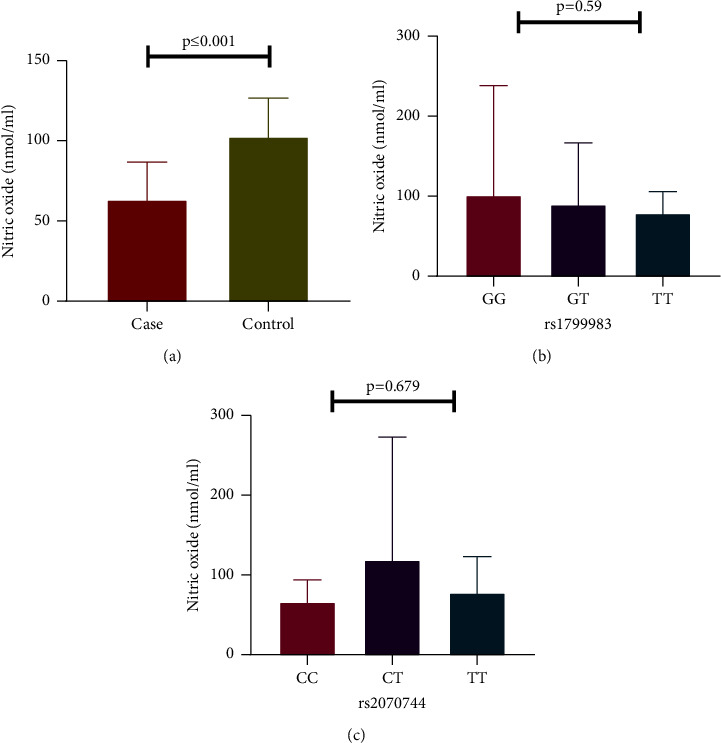
Circulating plasma NO levels between CKD cases and controls and the influence of *NOS3* polymorphisms (rs1799983 and rs2070744) on NO levels.

When CKD patients and controls were compared for plasma NO levels, they were found to be significantly decreased in CKD patients compared to the controls (63.42 nmol/mL (53.85–86.66) vs. 102.72 nmol/mL (79.70–126.52), *P* ≤ 0.001) (Figure 4). Genetic variations in *NOS3* gene (rs1799983 and rs2070744) were not correlated with stage of CKD, metabolic syndrome (Supplementary Tables 7 and 8), and plasma NO level (Figure 4). We found no significant correlation between MGP, NO, and FMD (Supplementary Table 9).

**Figure 5:**
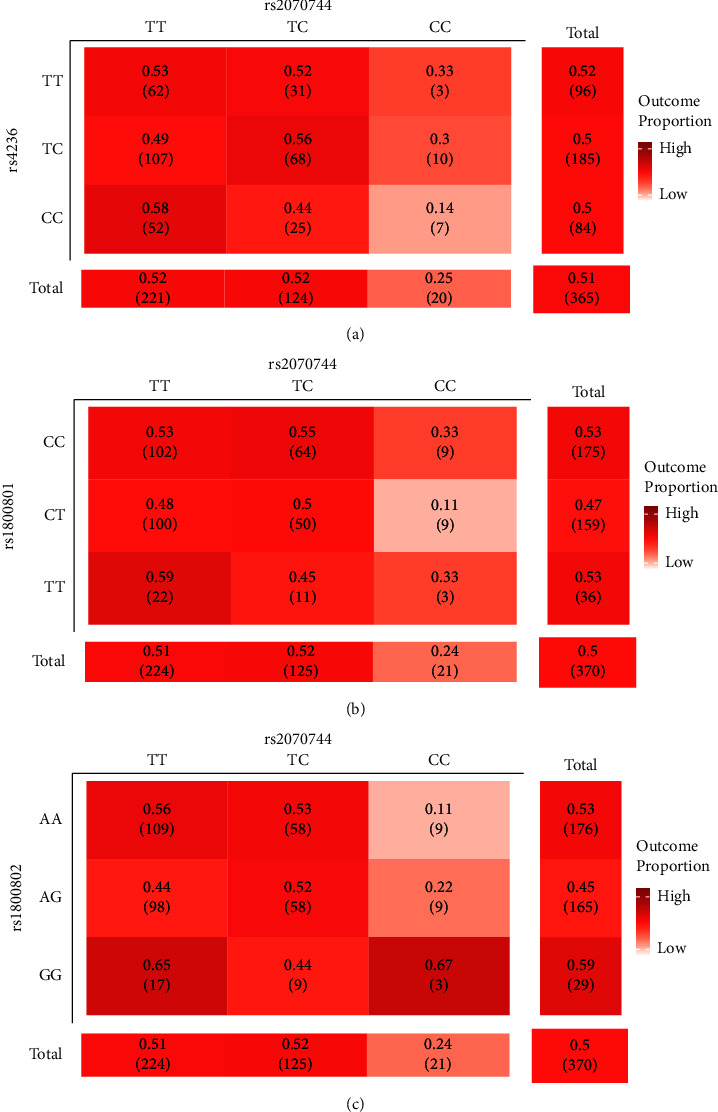
SNP-SNP interaction patterns associated with CKD. (a) rs4236 (*MGP*)-rs2070744 (*NOS3*) in CKD with the DR_int_oo_ interaction pattern. (b) rs1800801 (*MGP*)-rs2070744 (*NOS3*) in CKD with the RR_int_ro_ interaction pattern. (c) rs1800802 (*MGP*)-rs2070744 (*NOS3*) in CKD with the RR_int_ro_ interaction pattern.

When we checked for the SNP-SNP interactions between *MGP* and *NOS3* polymorphisms, we found a significant interaction between three SNP-SNP pairs (Table 7). The risk of developing CKD was reduced in those with the CC genotype of rs4236 (*MGP*) + CC genotype of rs2070744 (*NOS3*), OR = 0.26 (95% confidence interval [CI] = 0.08–0.83), *P*=0.02. People who carried the CT genotype of rs1800801 (*MGP*) + CC genotype of rs2070744 (*NOS3*) were less likely to develop CKD; OR = 0.26 (95% CI = 0.08–0.83), *P*=0.02. A decreased risk of developing CKD was seen in those with the AA genotype of rs1800802 (*MGP*) + CC genotype of rs2070744 (*NOS3*), OR = 0.18 (95% CI = 0.05–0.65), *P*=0.008 (Figure 5).

## 4. Discussion

In this study, we explored the association of *MGP* and *NOS3* polymorphisms with the susceptibility of CKD and its associated cardiovascular comorbidity in South Indian Tamils, and our data showed that TT and CT genotypes of *NOS3* (rs2070744) and TTA and CCG haplotypes of *MGP* (1-rs4236; 2-rs1800801; 3-rs1800802) genetic variants are associated with CKD risk in South Indian Tamils. The CT genotype of *MGP* (rs4236) polymorphism and CT genotype of *NOS3* (rs2070744) polymorphism were found to be associated with decreased FMD, indicating endothelial dysfunction, the harbinger of CVD.

We did not find any significant difference in the genotype and allele frequency of *MGP* rs1800801, rs1800802, and rs4236 polymorphisms between the two groups. However, The CT genotype of MGP (rs4236) had decreased FMD, indicating endothelial dysfunction. This differed from a study on CKD patients undergoing kidney transplants in a Swedish hospital. They found a significant difference in the rs1800801 CC genotype and rs4236 TT genotype between the cases and controls. However, they did not find any significant association between the genotypes and phenotypes of the disease, such as age, sex, circulating MGP levels, and coronary artery calcification scores [8]. In the Spanish population, rs4236 SNP was associated with an increased risk of CKD [18, 19]. Another retrospective study on hemodialysis patients in Japan by Yoshikawa et al. found that patients with the CC genotype of *MGP* rs1800802 experienced considerably slower vascular calcification progression than those with the CT or TT genotype [4]. In the Northern European population, *MGP* (rs1800801, rs1800802, and rs4236) SNPs significantly predict coronary artery calcification in men but not in females [20]. TT genotype of the *MGP* SNP rs1800801 is associated with stroke recurrence in the North American Caucasian population [21]. In CKD patients compared to controls, we found a statistically significant difference in the distribution of the TTA and CCG haplotypes (1-rs4236; 2-rs1800801; 3-rs1800802), indicating an increase in the risk of CKD.

Increased vascular and coronary artery calcification has been linked to decreased MGP levels. There are conflicting findings in the literature about the levels of MGP in CKD patients. Our study did not find any significant difference between the plasma levels of MGP between the CKD cases and controls. Few studies observed greater MGP levels in CKD patients than in controls, possibly due to reduced renal function [22, 23]. However, a study by Sevinc et al. [24] found a decreased level of MGP in CKD cases compared to the controls. We also did not find any impact of *MGP* polymorphism on the presence of metabolic syndrome and plasma MGP levels which was concordant with Jaminon et al. [8].

We explored the association of *NOS3* (rs1799983 and rs2070744) polymorphisms with the development of CKD and its associated clinical phenotype. We did not find any significant difference in the genotype frequency of *NOS3* rs1799983. It was contradictory to the studies conducted in other populations. Bambha et al. reported that the variant allele of *NOS3* SNP rs1799983 is linked to CKD incidence after liver transplantation and may help identify patients more likely to develop post-liver transplantation CKD [25]. In CKD patients, poorer left ventricular ejection fraction was associated with the *NOS3* rs1799983 polymorphism's GG genotype [26].

We found that the CC genotype of *NOS3* rs2070744 polymorphism is a significant protective factor against CKD development. When CC genotype of NOS3 rs2070744 was present with either CC genotype of rs4236 (MGP) or CT genotype of rs1800801 (MGP), or AA genotype of rs1800802 (MGP); there was a decrease in risk of developing CKD. The recessive model analysis revealed that TT and CT genotypes of *NOS3* rs2070744 were risk genotypes for CKD. Also, in our study, we found that the CT genotype of rs2070744 (NOS3) had decreased FMD, indicating endothelial dysfunction. A similar result was observed in the Egyptian population. A significant association between the TT and TC genotypes of *NOS3* (rs2070744) gene polymorphism with a higher incidence of ESRD and CKD was found [27]. According to another study conducted in a Malaysian population by Ahmad et al., the environmental factors and the *NOS3* rs2070744 polymorphism were found to alter the likelihood of developing CKD [28]. In contrast to our study, in a Spanish population of CKD patients with type 2 diabetes, the *NOS3* rs2070744's CC genotype was linked to advanced chronic kidney disease [16].

We discovered that the plasma NO levels are significantly lower in CKD cases than the controls. Our results were concordant with Schmidt and Baylis [29] and Reddy et al. [30] who also found a decreased NO level in CKD cases compared to controls which may be due to various factors such as oxidative stress, reduced L-arginine synthesis and transport, diversion of L-arginine through other metabolic pathways, failure of renal tubular L-arginine reclamation, and increase of NOS inhibitors like asymmetric dimethylarginine. In this study, we observed that *NOS3* polymorphism does not have any association with stage of CKD, presence of metabolic syndrome, as well as the level of plasma NO.

This study had a few limitations. Due to financial restraints, we could not measure the phosphorylated MGP and dephosphorylated-uncarboxylated MGP separately which would have helped us to understand the functional role of MGP in regulating calcification and its relation with polymorphisms. Because of ethical concerns related to the potential risks associated with radiation exposure, we were unable to access the direct measures of vascular calcifications, such as the CAC score. Studying more SNPs in the *MGP* and *NOS3* genes would have improved our understanding of how LD affects CKD and CVD susceptibility.

## 5. Conclusion

We conclude that genetic variants of *MGP* and *NOS3* enhance the risk of CKD and are associated with endothelial dysfunction, which predisposes to CVD. Expression of MGP and NOS3 at the genetic and epigenetic levels and their relation with CVD need to be investigated in future studies on CKD patients.

## Figures and Tables

**Table 1:** Comparison of baseline characteristics between cases and controls.

Parameter	Cases (*n* = 185) Median (IQR)	Controls (*n* = 185) Median (IQR)	*P* value
Age (years)	53.00 (45.00–62.00)	53.00 (45.50–60.00)	0.562
Gender (M:F)	140 : 45	140 : 45	—
BMI (kg/m^2^)	22.94 (20.01–25.44)	22.76 (20.83–24.22)	0.190
W/H ratio (W:H)	0.94 (0.87–0.99)	0.95 (0.93–0.97)	0.571
Systolic BP (mmHg)	126.00 (117.00–138.50)	113.00 (107.00–118.00)	**≤0.001**
Diastolic BP (mmHg)	83.00 (76.00–93.50)	73.00 (68.50–78.00)	**≤0.001**
eGFR (mL/min/1.73 m^2^)	28.00 (22.00–39.00)	—	
Duration of CKD (months)	43.00 (24.00–64.00)	—	
Family history of kidney disease (yes:no)	29 : 156	—	
Personal history of fracture (yes:no)	6 : 179	—	
Personal history of smoking (yes:no)	60 : 125	—	
Personal history of alcoholism (yes:no)	90 : 95	—	
Metabolic syndrome (yes:no)	26 : 159	—	

Mann–Whitney *U* test was used to analyze the data. Statistically significant *p* values are given in bold.

**Table 2:** Genotype and allele frequencies of *MGP* polymorphism in CKD cases and healthy controls.

Genotype/allele	Cases (*n* = 185)	Controls (*n* = 185)	*P* value	OR (95% CI)
rs1800801				
CC	92 (49.7%)	83 (44.9%)	—	—
CT	74 (40%)	85 (45.9%)	0.27	0.78 (0.50–1.21)
TT	19 (10.3%)	17 (9.2%)	0.99	1.00 (0.50–2.02)
C	258 (69.7%)	251 (67.8%)	—	—
T	112 (30.3%)	119 (32.2%)	0.57	0.91 (0.66–1.25)
rs1800802				
AA	93 (50.3%)	83 (44.9%)	—	—
AG	75 (40.5%)	90 (48.6%)	0.17	0.74 (0.48–1.13)
GG	17 (9.2%)	12 (6.5%)	0.68	1.26 (0.56–2.86)
A	261 (70.5%)	256 (69.2%)	—	—
G	109 (29.5%)	114 (30.8%)	0.68	0.93 (0.68–1.27)
rs4236				
TT	50 (27.0%)	46 (24.9%)	—	—
CT	93 (50.3%)	97 (52.4%)	0.61	0.88 (0.54–1.45)
CC	42 (22.7%)	42 (22.7%)	0.78	0.92 (0.51–1.64)
T	193 (52.2%)	189 (51.1%)	—	—
C	177 (47.8%)	181 (48.9%)	0.76	0.95 (0.71–1.28)

Fischer's exact test/chi‐square test was used to analyze the genotype and allele frequencies. *P* value < 0.05 was considered statistically significant.

**Table 3:** Association between *MGP* (rs1800801, rs1800802, and rs4236), *NOS3* polymorphisms (rs1799983 and rs2070744), and flow-mediated dilatation (FMD) of brachial artery.

rs1800801	CC	CT	TT	*P* value
FMD (<7-low)	4.26 (3.03–5.88)	5.00 (3.42–6.01)	3.75 (2.68–5.85)	0.329
FMD (>7-normal to high)	12.50 (10.44–20.00)	14.12 (10.04–20.00)	12.90 (10.53–14.29)	0.841

rs1800802	AA	AG	GG	*P* value

FMD (<7-low)	4.60 (2.96–6.06)	4.88 (3.64–5.88)	3.57 (2.56–4.76)	0.472
FMD (>7-normal to high)	13.04 (10.59–19.38)	12.50 (9.09–18.42)	13.34 (11.35–18.13)	0.506

rs4236	CC	CT	TT	*P* value

FMD (<7-low)	5.56 (4.29–6.50)	4.30 (2.63–5.19)	4.65 (3.40–6.06)	**0.041**
FMD (>7-normal to high)	12.70 (10.74–15.80)	13.89 (10.13–18.98)	12.50 (10.44–19.38)	0.987

rs1799983	GG	GT	TT	*P* value

FMD (<7-low)	4.76 (3.17–5.97)	4.45 (2.90–5.88)	3.66 (2.44–4.88)	0.574
FMD (>7-normal to high)	12.50 (10.17–17.91)	14.29 (10.53–20.26)	10.91 (8.92–13.89)	0.381

rs2070744	TT	CT	CC	*P* value

FMD (<7-low)	5.06 (3.57–6.25)	3.61 (2.56–4.88)	4.88 (4.26–6.25)	**0.01**
FMD (>7-normal to high)	13.04 (10.30–18.35)	13.89 (10.71–20.00)	13.06 (11.11–15.00)	0.834

Kruskal–Wallis test was used to analyze the genotype and allele frequencies. *P* value < 0.05 was considered statistically significant. Statistically significant *p* values are given in bold.

**Table 4:** Haplotype frequencies of *MGP* polymorphisms in CKD patients and controls.

Sl. no.	Haplotypes (1-2-3)	CKD (%) *N* = 185	Controls (%) *N* = 185	*P* value
1	TCG	0.244	0.286	0.19
2	CTA	0.235	0.286	0.11
3	TCA	0.211	0.192	0.50
4	CCA	0.197	0.190	0.80
5	TTA	0.062	0.025	**0.01**
6	CCG	0.045	0.011	**0.004**

1-rs4236; 2-rs1800801; 3-rs1800802. Statistically significant *p* values are given in bold.

**Table 5:** Genotype and allele frequencies of *NOS3* polymorphism in CKD cases and healthy controls.

Genotype/allele	Cases (*n* = 185)	Controls (*n* = 185)	*P* value	OR (95% CI)
rs1799983				
GG	130 (70.3%)	139 (75.1%)	—	—
GT	49 (26.5%)	39 (21.1%)	0.23	1.34 (0.82–2.14)
TT	6 (3.2%)	7 (3.8%)	0.99	0.91 (0.29–2.52)
T	61 (16.5%)	53 (14.3%)	—	—
G	309 (83.5%)	317 (85.7%)	0.41	0.84 (0.56–1.25)
rs2070744				
TT	115 (62.2%)	109 (58.9%)	—	—
CT	65 (35.1%)	60 (32.4%)	0.90	1.02 (0.65–1.57)
CC	5 (2.7%)	16 (8.7%)	**0.02**	0.29 (0.11–0.84)
T	295 (79.7%)	278 (75.1%)	—	—
C	75 (20.3%)	92 (24.9%)	0.13	0.76 (0.54–1.08)

Fischer's exact test/chi‐square test was used to analyze the genotype and allele frequencies. *P* value < 0.05 was considered statistically significant. Statistically significant *p* values are given in bold.

**Table 6:** Haplotype frequencies of *NOS3* polymorphisms in CKD patients and controls.

Sl. no.	Haplotypes (1-2)	CKD (%) *N* = 185	Controls (%) *N* = 185	*P* value
1	TG	0.674	0.66	0.69
2	CG	0.161	0.197	0.21
3	TT	0.124	0.091	0.15
4	CT	0.041	0.052	0.48

1-rs2070744; 2-rs1799983.

**Table 7:** SNP-SNP interaction between different genes in CKD cases.

SNP pair	Gene 1	Gene 2	Pattern	OR (95% CI)	*P* value
rs4236_rs1799983	*MGP*	*NOS3*	DR_int_ro	4.64*E* − 07 (0-inf)	0.977
rs4236_rs2070744	*MGP*	*NOS3*	DR_int_oo	0.26 (0.08–0.83)	**0.02**
rs1800801_rs1799983	*MGP*	*NOS3*	RR_int_oo	4.67*E* − 07 (0-inf)	0.98
rs1800801_rs2070744	*MGP*	*NOS3*	RR_int_ro	0.26 (0.08–0.83)	**0.02**
rs1800802_rs1799983	*MGP*	*NOS3*	RD_int_oo	4.67 (0.99–21.95)	0.05
rs1800802_rs2070744	*MGP*	*NOS3*	RR_int_ro	0.18 (0.05–0.65)	**0.008**

DR_int_ro: dominant-recessive interaction model with reverse-ordinal coding for SNPs; DR_int_oo: dominant-recessive interaction model with original-original coding for SNPs; RR_int_oo: recessive-recessive interaction model with original-original coding for SNPs; RR_int_ro: recessive-recessive interaction model with reverse-ordinal coding for SNPs; and RD_int_oo: recessive-dominant interaction model with original-original coding for SNPs. Statistically significant *p* values are given in bold.

## Data Availability

The data that support the findings of this study are available from the corresponding author, upon reasonable request.

## Supplementary Materials

Supplementary Materials The supplementary material contains tables showing routine parameters, genetic model analysis, genotype phenotype analysis of the polymorphisms studied, and correlation between study parameters.
