# Supplementary material for: Matrix Gla Protein and Nitric Oxide Synthase-3 Genetic Variants in Chronic Kidney Disease and Their Relation with Cardiovascular Risk
Source: Int J Nephrol. 2024 Aug 30;2024:3850055. doi: 10.1155/2024/3850055 (PMC11379508; doi:10.1155/2024/3850055)
Supplement: Supplementary Materials — The supplementary material contains tables showing routine parameters, genetic model analysis, genotype phenotype analysis of the polymorphisms studied, and correlation between study parameters. [file 3850055.f1.docx]

**Supplementary table 1: Routine parameters between CKD cases and controls**

| **Parameter** | **Cases (n=185)**  **Mean ± SD / Median (IQR)** | **Controls (n=185)**  **Mean ± SD / Median (IQR)** | **P value** |
| --- | --- | --- | --- |
| Fasting blood sugar (mg/dl) | 91.00 (83.00-96.00) | 91.00 (83.50-96.00) | 0.865 |
| Creatinine (mg/dl) | 2.32 (1.79-2.99) | 0.90 (0.80-1.0) | **≤0.001** |
| Urea (mg/dl) | 52.00 (43.50-65.50) | 20.00 (17.00-22.00) | **≤0.001** |
| Total cholesterol (mg/dl) | 167.00 (138.50-196.50) | 150.00 (132.50-163.50) | **≤0.001** |
| Triglycerides (mg/dl) | 119.00 (99.00-177.00) | 102.00 (74.00-135.00) | **≤0.001** |
| High density lipoprotein (mg/dl) | 39.00 (31.00-47.50) | 32.00 (28.00-37.00) | **≤0.001** |
| Low density lipoprotein (mg/dl) | 107.00 (84.00-133.00) | 93.00 (76.20-103.00) | **≤0.001** |
| Very low density lipoprotein (mg/dl) | 24.40 (20.00-36.00) | 20.00 (15.00-27.00) | **≤0.001** |
| Calcium (mg/dl) | 9.21 (8.91-9.58) | 9.4 (9.0-9.9) | **0.035** |
| Phosphorous (mg/dl) | 3.52 (3.06-4.11) | 3.20 (2.65-3.70) | **≤0.001** |
| Alkaline phosphatase (IU/L) | 96.00 (80.00-127.00) | 78.00 (62.00-101.50) | **≤0.001** |
| Uric acid (mg/dl) | 7.5 (6.43-8.81) | 5.2 (4.3-6.1) | **≤0.001** |
| Total protein (mg/dl) | 7.4 (7.08-7.64) | 7.6 (7.2-7.8) | **0.002** |
| Albumin (mg/dl) | 4.2 (4.0-4.4) | 4.3 (4.1-4.5) | **≤0.001** |
| Flow mediated dilatation of brachial artery (%) | 10.34 (5.79-14.90) | 40.91 (36.09-44.95) | **≤0.001** |

Mann-Whitney U test was used to compare the groups

**Supplementary table 2: Genetic model analysis for *MGP* polymorphism in CKD cases and healthy controls**

| **SNP (rsID)** | **Model** | **Genotype** | **Cases**  **(*n* = 185)** | **Controls**  **(*n* = 185)** | ***p* value** | **OR (95% CI)** |
| --- | --- | --- | --- | --- | --- | --- |
| **rs1800801** | **Dominant**  **Recessive**  **Co-dominant**  **Homozygotic** | CT + TT  CC  CT + CC  TT  TT + CC  CT  TT  CC | 93 (50.3%)  92 (49.7%)  166 (89.7%)  19 (10.3%)  111 (60%)  74 (40%)  112 (30.3%)  258 (69.7%) | 102 (55.1%)  83 (44.9%)  168 (90.8%)  17 (9.2%)  100 (54.1%)  85 (45.9%)  119 (32.2%)  251 (67.8%) | 0.34  -  0.86  -  0.24  -  0.57  - | 0.82 (0.54-1.24)  -  0.88 (0.43-1.76)  -  1.27 (0.83-1.90)  -  0.91 (0.66-1.25)  - |
| **rs1800802** | **Dominant**  **Recessive**  **Co-dominant**  **Homozygotic** | AG + GG  AA  AA + AG  GG  AA + GG  AG  GG  AA | 92 (49.7%)  93 (50.3%)  168 (90.8%)  17 (9.2%)  110 (59.5%)  75 (40.5%)  109 (29.5%)  261 (70.5%) | 102 (55.1%)  83 (44.9%)  173 (93.5%)  12 (6.5%)  95 (51.4%)  90 (48.6%)  114 (30.8%)  256 (69.2%) | 0.29  -  0.43  -  0.11  -  0.68  - | 0.80 (0.53-1.21)  -  0.68 (0.31-1.46)  -  1.38 (0.91-2.07)  -  0.93 (0.68-1.27)  - |
| **rs4236** | **Dominant**  **Recessive**  **Co-dominant**  **Homozygotic** | CC + CT  TT  CT + TT  CC  CC + TT  CT  CC  TT | 135 (73.0%)  50 (27.0%)  143 (77.3%)  42 (22.7%)  92 (49.7%)  93 (50.3%)  177 (47.8%)  193 (52.2%) | 139 (75.1%)  46 (24.9%)  143 (77.3%)  42 (22.7%)  88 (47.6%)  97 (52.4%)  181(48.9%)  189 (51.1%) | 0.63  -  0.99  -  0.67  -  0.76  - | 0.89 (0.56-1.40)  -  1.00 (0.60-1.64)  -  1.09 (0.72-1.64)  **-**  0.95 (0.71-1.28)  **-** |

Fischer's exact test/chi‐square test was used to analyse the genotype and allele frequencies. *p* value < 0.05 was considered statistically significant.

**Supplementary table 3: Influence of *MGP* (rs1800801) genotype on the phenotypes of CKD**

| **Phenotype** | **MGP gene (rs1800801)** | **Cases with phenotype**  **(*n*)** | **Cases without**  **phenotype (*n*)** | ***p* value** | **OR (95% CI)** |
| --- | --- | --- | --- | --- | --- |
| Female | CC Vs CT + TT  TT  CT  CC | 22  4  18  23 | 71  15  56  69 | 0.83  0.99  0.99  - | 0.92 (0.48-1.78)  0.80 (0.26-2.39)  0.96 (0.48-1.97)  - |
| Family History of kidney disease | CC Vs CT + TT  TT  CT  CC | 16  6  10  13 | 77  13  64  79 | 0.68  0.09  0.99  - | 1.26 (0.54-2.78)  2.80 (0.85-8.47)  0.94 (0.38-2.40)  - |
| CKD stage 4 | CC Vs CT + TT  TT  CT  CC | 48  10  38  52 | 45  9  36  40 | 0.50  0.80  0.50  - | 0.82 (0.46-1.44)  0.85 (0.33-2.20)  0.81 (0.44-1.47)  - |
| CKD stage 3 | CC Vs CT + TT  TT  CT  CC | 41  8  33  38 | 52  11  41  54 | 0.70  0.99  0.67  - | 1.12 (0.63-2.0)  1.03 (0.37-2.92)  1.14 (0.62-2.08)  - |
| CKD stage 2 | CC Vs CT + TT  TT  CT  CC | 4  1  3  2 | 89  18  71  90 | 0.68  0.43  0.65  - | 2.02 (0.46-10.81)  2.5 (0.16-22.14)  1.90 (0.37-10.90)  - |
| Metabolic syndrome | CC Vs CT + TT  TT  CT  CC | 11  3  8  13 | 82  16  66  79 | 0.66  0.99  0.64  - | 0.81 (0.35-1.97)  1.13 (0.31-4.41)  0.73 (0.29-1.78)  - |

Fischer's exact test/chi‐square test was used to analyse association of genotype and phenotype. *p* value < 0.05 was considered statistically significant.

**Supplementary table 4: Influence of *MGP* (rs1800802) genotype on the phenotypes of CKD**

| **Phenotype** | **MGP gene (rs1800802)** | **Cases with phenotype (*n*)** | **Cases without**  **phenotype (*n*)** | ***p* value** | **OR (95% CI)** |
| --- | --- | --- | --- | --- | --- |
| Female | AA Vs AG + GG  GG  AG  AA | 22  5  17  23 | 70  12  58  70 | 0.12  0.76  0.85  - | 0.95 (0.49-1.83)  1.26 (0.45-3.71)  0.89 (0.44-1.84)  - |
| Family History of kidney disease | AA Vs AG + GG  GG  AG  AA | 12  5  7  17 | 80  12  68  76 | 0.41  0.32  0.12  - | 0.67 (0.29-1.53)  1.86 (0.64-5.77)  0.46 (0.18-1.16)  - |
| CKD stage 4 | AA Vs AG + GG  GG  AG  AA | 55  9  46  45 | 37  8  29  48 | 0.11  0.79  0.09  - | 1.58 (0.89-2.84)  1.2 (0.43-3.49)  1.69 (0.89-3.11)  - |
| CKD stage 3 | AA Vs AG + GG  GG  AG  AA | 35  7  28  44 | 57  10  47  49 | 0.20  0.79  0.19  - | 0.68 (0.37-1.21)  0.77 (0.29-2.08)  0.66 (0.35-1.25)  - |
| CKD stage 2 | AA Vs AG + GG  GG  AG  AA | 2  1  1  4 | 90  16  74  89 | 0.68  0.57  0.38  - | 0.49 (0.09-2.17)  1.39 (0.10-9.46)  0.30 (0.02-1.88)  - |
| Metabolic syndrome | AA Vs AG + GG  GG  AG  AA | 9  1  8  15 | 83  16  67  78 | 0.27  0.45  0.37  - | 0.56 (0.23-1.35)  0.32 (0.02-2.03)  0.62 (0.25-1.57)  - |

Fischer's exact test/chi‐square test was used to analyse association of genotype and phenotype. *p* value < 0.05 was considered statistically significant.

**Supplementary table 5: Influence of *MGP* (rs4236) genotype on the phenotypes of CKD**

| **Phenotype** | **MGP gene**  **(rs4236)** | **Cases with phenotype (*n*)** | **Cases without**  **phenotype (*n*)** | ***p* value** | **OR (95% CI)** |
| --- | --- | --- | --- | --- | --- |
| Female | TT Vs TC + CC  CC  TC  TT | 31  5  26  14 | 104  37  67  36 | 0.56  0.07  0.99  - | 0.76 (0.36-1.58)  0.34 (0.12-1.08)  0.99 (0.47-2.08)  - |
| Family History of kidney disease | TT Vs TC + CC  CC  TC  TT | 17  6  11  12 | 118  36  82  38 | 0.06  0.29  0.09  **-** | 0.45 (0.20-1.09)  0.52 (0.17-1.50)  0.42 (0.17-1.01)  - |
| CKD stage 4 | TT Vs TC + CC  CC  TC  TT | 69  18  51  31 | 66  24  42  19 | 0.18  0.09  0.40  - | 0.64 (0.32-1.24)  0.45 (0.19-1.02)  0.74 (0.38-1.48)  - |
| CKD stage 3 | TT Vs TC + CC  CC  TC  TT | 62  21  41  17 | 73  21  52  33 | 0.18  0.14  0.28  - | 1.64 (0.83-3.17)  1.94 (0.85-4.58)  1.53 (0.75-3.09)  - |
| CKD stage 2 | TT Vs TC + CC  CC  TC  TT | 4  3  1  2 | 131  39  92  48 | 0.66  0.65  0.27  - | 0.73 (0.16-3.95)  1.84 (0.36-10.73)  0.26 (0.01-56.05)  - |
| Metabolic syndrome | TT Vs TC + CC  CC  TC  TT | 16  7  9  8 | 119  35  84  42 | 0.46  0.99  0.28  - | 0.70 (0.28-1.73)  1.05 (0.37-3.17)  0.56 (0.21-1.60)  - |

Fischer's exact test/chi‐square test was used to analyse association of genotype and phenotype. *p* value < 0.05 was considered statistically significant.

**Supplementary table 6: Genetic model analysis for *NOS3* polymorphisms in CKD cases and healthy controls**

| **SNP (rsID)** | **Model** | **Genotype** | **Cases**  **(*n* = 185)** | **Controls**  **(*n* = 185)** | ***p* value** | **OR (95% CI)** |
| --- | --- | --- | --- | --- | --- | --- |
| **rs1799983** | **Dominant**  **Recessive**  **Co-dominant**  **Homozygotic** | TT + GT  GG  GG + GT  TT  GG + TT  GT  GG  TT | 55 (29.7%)  130 (70.3%)  179 (96.8%)  6 (3.2%)  136 (73.5%)  49 (26.5%)  309 (83.5%)  61 (16.5%) | 46 (24.9%)  139 (75.1%)  178 (96.2%)  7 (3.8%)  146 (78.9%)  39 (21.1%)  317 (85.7%)  53 (14.3%) | 0.29  -  0.99  -  0.22  -  0.41  - | 1.27 (0.80-1.99)  -  1.17 (0.42-3.59)  -  0.74 (0.45-1.19)  -  0.84 (0.56-1.25)  - |
| **rs2070744** | **Dominant**  **Recessive**  **Co-dominant**  **Homozygotic** | CC +CT  TT  TT + CT  CC  TT +CC  CT  CC  TT | 70 (37.8%)  115 (62.2%)  180 (97.3%)  5 (2.7%)  120 (64.9%)  65 (35.1%)  75 (20.3%)  295 (79.7%) | 76 (41.1%)  109 (58.9%)  169 (91.3%)  16 (8.7%)  125 (67.6%)  60 (32.4%)  92 (24.9%)  278 (75.1%) | 0.52  -  **0.02**  -  0.58  -  0.13  - | 0.87 (0.57-1.31)  -  3.40 (1.22-8.61)  -  0.88 (0.57-1.37)  -  0.76 (0.54-1.08)  - |

Fischer's exact test/chi‐square test was used to analyse the genotype and allele frequencies. *p* value < 0.05 was considered statistically significant. All significant results are in bold font.

**Supplementary table 7: Influence of *NOS3* (rs1799983) genotype on the phenotypes of CKD**

| **Phenotype** | **NOS3 gene (rs1799983)** | **Cases with phenotype (*n*)** | **Cases without**  **phenotype (*n*)** | ***p* value** | **OR (95% CI)** |
| --- | --- | --- | --- | --- | --- |
| Female | GG Vs GT + TT  TT  GT  GG | 13  2  11  32 | 42  4  38  98 | 0.99  0.63  0.84  - | 0.94 (0.46-1.97)  1.53 (0.28-6.80)  0.88 (0.40-1.97)  - |
| Family History of kidney disease | GG Vs GT + TT  TT  GT  GG | 12  0  12  17 | 43  6  37  113 | 0.18  0.99  0.07  **-** | 1.85 (0.78-3.99)  0.00 (0.00-4.12)  2.15 (0.89-4.73)  - |
| CKD stage 4 | GG Vs GT + TT  TT  GT  GG | 31  5  26  69 | 24  1  23  61 | 0.68  0.21  0.99  - | 1.14 (0.61-2.18)  4.42 (0.57-52.84)  0.99 (0.50-1.88)  - |
| CKD stage 3 | GG Vs GT + TT  TT  GT  GG | 23  1  22  56 | 32  5  27  74 | 0.87  0.40  0.82  - | 0.94 (0.49-1.78)  0.26 (0.02-2.04)  1.07 (0.56-2.12)  - |
| CKD stage 2 | GG Vs GT + TT  TT  GT  GG | 1  0  1  5 | 54  6  48  125 | 0.67  0.99  0.99  - | 0.46 (0.03-3.52)  0.00 (0.00-19.57)  0.52 (0.04-3.98)  - |
| Metabolic syndrome | GG Vs GT + TT  TT  GT  GG | 7  0  7  17 | 48  6  42  113 | 0.99  0.99  0.83  - | 0.96 (0.38-2.47)  0.00 (0.00-4.12)  1.10 (0.43-2.86)  - |

Fischer's exact test/chi‐square test was used to analyse association of genotype and phenotype. *p* value < 0.05 was considered statistically significant.

**Supplementary table 8: Influence of *NOS 3* (rs2070744) genotype on the phenotypes of CKD**

| **Phenotype** | **NOS3 gene (rs2070744)** | **Cases with phenotype (*n*)** | **Cases without**  **Phenotype (*n*)** | ***p* value** | **OR (95% CI)** |
| --- | --- | --- | --- | --- | --- |
| Female | TT Vs TC + CC  CC  TC  TT | 16  1  15  29 | 54  4  50  86 | 0.85  0.99  0.85  - | 0.87 (0.44-1.72)  0.74 (0.05-4.81)  0.88 (0.43-1.78)  - |
| Family History of kidney disease | TT Vs TC + CC  CC  TC  TT | 14  2  12  15 | 56  3  53  100 | 0.20  0.14  0.38  - | 1.66 (0.76-3.56)  4.44 (0.72-22.8)  1.50 (0.63-3.37)  - |
| CKD stage 4 | TT Vs TC + CC  CC  TC  TT | 39  3  36  61 | 31  2  29  54 | 0.72  0.99  0.76  - | 1.11 (0.61-2.05)  1.32 (0.26-7.68)  1.09 (0.58-1.98)  - |
| CKD stage 3 | TT Vs TC + CC  CC  TC  TT | 29  2  27  50 | 41  3  38  65 | 0.78  0.99  0.80  - | 0.91 (0.51-1.68)  0.86 (0.14-4.38)  0.92 (0.50-1.74)  - |
| CKD stage 2 | TT Vs TC + CC  CC  TC  TT | 2  0  2  4 | 68  5  63  111 | 0.99  0.99  0.99  - | 0.81 (0.15-3.58)  0.00 (0.00-34.29)  0.88 (0.16-3.87)  - |
| Metabolic syndrome | TT Vs TC + CC  CC  TC  TT | 9  1  8  15 | 61  4  57  100 | 0.99  0.51  0.99  - | 0.98 (0.41-2.37)  1.66 (0.12-11.39)  0.93 (0.38-2.36)  - |

Fischer's exact test/chi‐square test was used to analyse association of genotype and phenotype. *p* value < 0.05 was considered statistically significant.

**Supplementary table 9: Correlation of Matrix gla protein, Nitric oxide and flow mediated dilatation**

|  | **Nitic oxide** | | **Matrix gla protein** | | **FMD** | |
| --- | --- | --- | --- | --- | --- | --- |
|  | **Rho value** | **p-value** | **Rho value** | **p-value** | **Rho value** | **p-value** |
| **Nitric oxide** | - | - | 0.059 | 0.610 | 0.205 | 0.075 |
| **Matrix gla protein** | 0.059 | 0.610 | - | - | 0.083 | 0.477 |
| **FMD** | 0.205 | 0.075 | 0.083 | 0.477 | - | - |

Spearman rank correlation test
